# P245: Descriptive analysis of measles cases in an universitary hospital and its health area

**DOI:** 10.1186/2047-2994-2-S1-P245

**Published:** 2013-06-20

**Authors:** S Justo Gil, T Gimenez-Julvez, CA De la Hoz-Gonzalez, A Gimenez-Cabrera, V Muñoz-Sanz, E Rodriguez-Baena

**Affiliations:** 1 Hospital Universitario Infanta Leonor, Spain; 2 Sureste Public Health Area, Madrid, Spain

## Introduction

Measles is a leading cause of vaccine-preventable childhood mortality. Despite having a vaccine with a protective efficacy of 95%, outbreaks are possible if vaccination coverage is below 95%. In our area there are irregular settlements where outbreaks can occur. The objetive of this paper is to describe the most relevant data of reported cases in our hospital and compare them with our health area data.

## Objectives

The objetive is to describe the most relevant data of reported cases in our hospital and compare them with our health area data.

## Methods

Registration in the Public Health Information System of reported cases, age, sex, serological status, hospital admission, measles vaccination status and risk group from March, 1^st^ 2009 to September, 1^st^ 2011. Data were analyzed globally and by epidemiological weeks.

## Results

Our hospital notified 28 (33.3%) suspicious cases of the total area (84). Median and interquartile (IQR) age 6 years (0.75 -18.5) versus 7.5 (1.5-18.3) in the area. Both modes were less than 1 year. 60.7% females in our hospital versus 58.3% females in the area. Confirmed serology hospital cases were 22 (78.6%) versus 56 (66.7%) area cases. 46,4% needed hospital admission in our cases, 25% in the area.

96.4% of the hospital cases were not vaccinated versus 92.9% in the area. 67% were from risk groups (irregular settlements and gypsy ethnic group) versus 58.3% of total area.

Incidence peak in our hospital was between 30 and 34 epidemiological weeks, later than our area (27-34 epidemiological weeks).

## Conclusion

Despite appropriate vaccination coverage in the Community of Madrid, outbreaks had occurred in groups of susceptible individuals (marginal communities and children under one year who have not been vaccinated). So in 2011, the Community of Madrid came early the first-dose of measles vaccine from 15 to 12 months. Since mode is less than one year, it would be appropriate to come early the first-dose of measles vaccine between six months and a year.

## Disclosure of interest

None declared

